# Medical adverse events in the US 2018 mortality data

**DOI:** 10.1016/j.pmedr.2021.101574

**Published:** 2021-10-02

**Authors:** Petteri Oura

**Affiliations:** a Department of Forensic Medicine, Faculty of Medicine, University of Helsinki, P.O. Box 21 (Haartmaninkatu 3), FI-00014, Helsinki, Finland; b Forensic Medicine Unit, Finnish Institute for Health and Welfare, P.O. Box 30 (Mannerheimintie 166), FI-00271, Helsinki, Finland; c Center for Life Course Health Research, Faculty of Medicine, University of Oulu, P.O. Box 5000, FI-90014, Oulu, Finland

**Keywords:** Adverse event, Medical error, Epidemiology, Mortality, US, National Vital Statistics

## Abstract

Although medical error has been estimated as a major cause of death in the US, the capability of current diagnostic coding systems and standard death certificates to capture these events has been criticized. This register-based study aimed to scrutinize medical adverse event deaths (i.e., deaths due to adverse events occurring within the healthcare practice, avoidable or unavoidable, including late complications and sequelae of such events) in the US National Vital Statistics 2018 mortality dataset. Individual-level data on underlying and multiple causes of death according to the tenth revision of the International Classification of Diseases (ICD-10) coding system were extracted together with the decedents’ sex, age, ethnicity and education level. Adverse event deaths were identified by ICD-10 codes Y40–Y84 and Y88. The dataset comprised a total of 2 846 305 certified deaths. An adverse event ICD-10 code was used as the underlying cause of death in 0.16% (n = 4620) of the cases, and appeared on the list of multiple causes in 1.13% (n = 32 226) of the cases. Odds for adverse event death were higher among younger than elderly individuals, among those of black than white ethnicity, and among individuals with higher education level. The present data indirectly support previous evidence that a large number of adverse events remain underrecognized or misclassified. Future analyses are needed to reveal the root causes behind underreporting and to analyze whether it occurs at random or in a systematic way.

## 1 Introduction

Medical adverse event is defined as an “unintended injury to patients caused by medical management” (Grober and Bohnen, 2005). While some adverse events are attributed to medical error (i.e., “the failure of a planned action to be completed as intended, or the use of a wrong plan to achieve an aim” (Kohn et al., 1999)) and are thus considered avoidable, others are due to factors that are not preventable (Garrouste-Orgeas et al., 2012). In the context of this study, the term “adverse event death” refers to deaths due to adverse events occurring within the healthcare practice, regardless of whether they are avoidable or unavoidable, including late complications and sequelae of such events.

In the majority of the world, causes of death are communicated using International Classification of Diseases (ICD) codes. Severe inaccuracies and underreporting are expected follow when a death is attributed to causes that are not well covered by the coding system, such as human and system factors (Makary and Daniel, 2016). As results obtained from standardized and reliable datasets should form the basis of future actions, investments in the research and prevention of adverse events and medical error have been urged (Kohn et al., 1999, Landrigan et al., 2010, Classen et al., 2011, Bogner, 1994, Schwendimann et al., 2018, Rafter et al., 2015, Wu et al., 2020). The topic is of high importance due to the number of patients and healthcare professionals affected, the problems aroused by such errors, and the subsequent societal impact (Bogner, 1994).

An analysis by Makary and Daniel in 2016 reviewed scientific literature on US deaths due to medical error, aiming to estimate their contribution to annual deaths. The data of four previous reports on preventable inpatient deaths from the years 2000–2008 were pooled and extrapolated to 2013, arriving at a striking point estimate of 251 454 annual deaths due to medical error (9.7% of all US deaths). Against the official cause-of-death statistics (Xu et al., 2016), this would translate as the third leading cause of death in the US, after heart disease and cancer. Makary and Daniel emphasized that their calculation was likely underestimating the full underlying mortality, as the pooled data included only medical errors that could be identified from health records, were considered preventable, and occurred in inpatient care.

Obviously, attempts to reduce adverse events and death from medical care should be based on reliable data. As there are concerns whether the current ICD coding and standard death certificates are able to efficiently capture adverse event deaths, this retrospective register-based study aimed to scrutinize these deaths in the US 2018 cause-of-death database. Data on a total of 2 846 305 certified deaths were retrieved from the database, including underlying and multiple causes of death as well as the decedents’ sex, age, ethnicity and education level. The frequency and general characteristics of adverse event deaths were presented and compared to those of non-adverse event deaths.

## 2 Materials and methods

### 2.1 Dataset

The dataset used in this study was retrieved from the US National Vital Statistics Online Data Portal (National Center for Health Statistics, Centers for Disease Control and Prevention, US), an intergovernmental database that disseminates official vital statistics collected by legal authorities (National Center for Health Statistics, 2020a). The database includes publicly available individual-level data to be used for statistical reporting and analysis under specified terms and conditions. For this study, the ”Mortality Multiple Cause US Data 2018″ package was retrieved in November 2020, containing all certified US deaths during the year of 2018, i.e., the most recent annual release available. As the present study was retrospective, registry-based, and utilized a public dataset, approval from ethics committee was not necessary.

### 2.2 Causes of death

The underlying (i.e., primary) and multiple causes of death were communicated to the database via death certificates (National Center for Health Statistics, 2020b) according to the tenth revision of the ICD coding system (ICD-10) (World Health Organization,). Up to 20 multiple causes were covered for each case in the dataset. Deaths due to an adverse event, including late complications and sequelae of such events, were proxied by the following ICD-10 codes: any adverse event, codes Y40—Y84 and Y88; medical or surgical misadventure, codes Y60—Y69 and Y88.1; complication of medical or surgical procedure without misadventure, codes Y83—Y84 and Y88.3; medication-related adverse event, codes Y40—Y59 and Y88.0; device-related adverse event, codes Y70—Y82 and Y88.2. As the primary approach, the underlying cause of death was used to discriminate adverse event deaths from non-adverse event deaths. As a secondary approach, the selection criteria were extended to cover the list of multiple causes (Redelings et al., 2006). In the multiple-cause data, analysis was based on unique death cases, with one adverse event code assigned to each case (i.e., the code with the smallest ordinal in the list of multiple causes).

### 2.3 Sociodemographics

Major sociodemographic traits (i.e., sex, age, ethnicity and educational history) were retrospectively reported to the database via death certificates (National Center for Health Statistics, 2020a, National Center for Health Statistics, 2020b). The variables were as follows: Sex was categorized as female/male. Age was grouped as < 20, 20–39, 40–59, 60–79, and ≥ 80 years. Ethnicity was grouped as white/black/others. Education level was categorized as high (i.e., bachelor’s degree or higher), medium (i.e., high school graduate or General Educational Development tests completed), and low (i.e., others).

### 2.4 Statistical analysis

The frequency (n) and proportion of adverse event deaths relative to all deaths (%) were calculated. The sociodemographic characteristics were presented using percentages and frequencies. The amount of missing data was low (unknown education, 1.8% of the dataset; unknown age, <0.1% of the dataset). Adverse event deaths were compared to non-adverse event deaths, and adverse event death subtypes were compared to each other, using multivariable logistic regression models which included the following variables: sex, age, ethnicity and education level. Exponentiated regression coefficients, i.e., odds ratios (ORs) were extracted with their 95% confidence intervals (CIs) and the associated P values. IBM SPSS Statistics version 26 (IBM, Armonk, NY, USA) was used to perform the statistical analysis. The threshold for statistical significance was set at P = 0.05.

## 3 Results

The dataset comprised a total of 2 846 305 certified deaths. An adverse event ICD-10 code was recorded on a death certificate a total of 32 400 times, was used as the underlying cause of death in 0.16% of the cases (4620/2 846 305), and appeared on the list of multiple causes in 1.13% of the cases (32 226/2 846 305) (Table 1). Of adverse event subtypes, procedure complication codes were the most common (87.2%; 4030/4620), followed by medication-related adverse events (10.9%; 503/4620) and deaths coded as medical and surgical misadventures (1.9%; 87/4620). There were no device-related adverse events in the underlying-cause dataset (Table 1).

**Table 1:** Distribution of adverse events in the US 2018 cause-of-death data.

Adverse event subtype	Underlying-cause data^1^			Multiple-cause data^2^		
	Frequency (n)	Proportion relative to all deaths (%)	Proportion relative to adverse event deaths (%)	Frequency (n)	Proportion relative to all deaths (%)	Proportion relative to unique adverse event deaths (%)
Any adverse event	4620	0.16	–	32 226/32 400^3^	1.13/1.14^3^	–
Complication of medical or surgical procedure without misadventure	4030	0.14	87.2	25 996	0.91	80.1
Medication-related adverse event	503	0.02	10.9	5983	0.21	18.6
Medical or surgical misadventure	87	<0.01	1.9	407	0.01	1.3
Medical device-related adverse event	0	0	0	14	<0.01	<0.1

1 Adverse event as the underlying cause of death.

2 Adverse event appearing on the list of multiple causes of death; one case may include several adverse event codes.

3 Presented as unique cases/total cases.

Table 2 presents sociodemographic characteristics of adverse event deaths and non-adverse event deaths. Odds for adverse event death were higher among younger than elderly individuals, among those of black than white origin, and among those with high education level (Table 2). A breakdown of sociodemographic characteristics by adverse event subtype is given in Supplementary Table 1.

**Table 2:** Sociodemographic characteristics of adverse event deaths with comparison to non-adverse event deaths according to underlying-cause data. Values are percentages with frequencies unless otherwise indicated.

Characteristic	All adverse event deaths (n = 4620)	All non-adverse event deaths (n = 2 841 685)	Comparison between adverse events and non-adverse events	
			OR^1^ (95% CI), P value	
Sex				
Female	46.6 (2152)	48.6 (1 380 868)	1 (Reference)	
Male	53.4 (2468)	51.4 (1 460 817)	0.97 (0.91–1.03)	0.303
Age (years)				
<20	2.0 (93)	1.5 (41 248)	**2.60 (2.08**–**3.26)**	**< 0.001**
20–39	4.0 (1 8 3)	4.1 (116 791)	**1.57 (1.34**–**1.84)**	**< 0.001**
40–59	16.9 (7 8 3)	13.0 (369 601)	**2.12 (1.94**–**2.33)**	**< 0.001**
60–79	50.3 (2323)	38.0 (1 080 182)	**2.16 (2.02**–**2.32)**	**< 0.001**
≥80	26.8 (1238)	43.4 (1 233 560)	1 (Reference)	
Unknown	0 (0)	<0.1 (3 0 3)		
Ethnicity				
White	81.8 (3779)	84.3 (2 394 238)	1 (Reference)	
Black	14.8 (6 8 4)	12.3 (349 147)	**1.13 (1.04**–**1.23)**	**0.005**
Other	3.4 (1 5 7)	3.5 (98 300)	0.98 (0.83–1.15)	0.761
Education level				
High	19.5 (9 0 0)	16.9 (480 698)	1 (Reference)	
Medium	61.9 (2860)	61.3 (1 742 006)	**0.84 (0.78**–**0.91)**	**< 0.001**
Low	17.0 (7 8 5)	20.0 (567 704)	**0.72 (0.65**–**0.79)**	**< 0.001**
Unknown	1.6 (75)	1.8 (51 277)		

CI = Confidence interval, OR = Odds ratio.

1 OR for adverse event death relative to non-adverse event death.

## 4 Discussion

This register-based study of 2 846 305 deaths aimed to scrutinize medical adverse event deaths in the US 2018 mortality database. The main finding was that only 1.13% of the deaths were attributed to an adverse event as the primary or contributory cause, and no more than 0.16% of all deaths were primarily attributed to an adverse event.

Reliable datasets with maximal coverage should be the first step to inform decision-making in attempts to reduce adverse events and death from medical care (Kohn et al., 1999, Landrigan et al., 2010, Classen et al., 2011, Bogner, 1994, Schwendimann et al., 2018, Rafter et al., 2015, Wu et al., 2020). However, there are concerns whether adverse events are efficiently captured as the cause of death in today’s practice. A recent analysis (Makary and Daniel, 2016) pooled data from the US to estimate that up to 251 454 deaths (9.7% of all deaths) occurred due to medical error in 2013. However, this estimate only addressed inpatient deaths due to medical error, making its comparison to the present analysis of adverse event deaths (0.16–1.13% of all deaths) potentially misleading. As the present approach was ICD-10 code-based, not limited to inpatient deaths and preventable causes, and used a relatively broad definition for adverse event death, the present figures would be expected to potentially exceed those of Makary and Daniel. Yet, they fall significantly short, indirectly supporting previous evidence that a substantial number of adverse events remain underrecognized (Classen et al., 2011). Most importantly, the present findings underline the urgent need to improve the documentation of deaths due to an adverse event (Kohn et al., 1999, Landrigan et al., 2010, Classen et al., 2011, Bogner, 1994, Schwendimann et al., 2018, Rafter et al., 2015, Wu et al., 2020).

The causes of underrecognition remain under speculation and require further study (Classen et al., 2011, Garrouste-Orgeas et al., 2012, Landrigan et al., 2010, Schwendimann et al., 2018, Rafter et al., 2015). A concern has been expressed as to whether the diagnostic coding systems have been designed to efficiently and accurately capture adverse events, and whether the standard death certificates have enough facility for acknowledging human and system factors as the cause of death (Makary and Daniel, 2016). To overcome this barrier, known or suspected adverse events preceding the death could be enquired as a separate item of the death certificate form, independently of the problematic ICD-10 codes. It would also seem crucial to quickly establish a reliable and centralized database for reporting all suspected and known adverse events in sufficient detail for research and public health purposes. Nevertheless, future studies should aim to identify the root causes behind underreporting and also address the aspect of whether it occurs at random or in a systematic way.

In this dataset, most adverse event deaths occurred among individuals who were male, aged 60–79 years, white, and had medium-level education. In light of the multivariable analysis, odds for adverse event death were higher among younger than elderly age groups, among individuals of black than white ethnicity, and among those with higher education level. Unfortunately, as this study was based on a mortality dataset with only the endpoint (i.e., death) and limited retrospectively collected sociodemographic data available, there were a number of potential confounders that could not be addressed. A prospective cohort design with detailed data on sociodemographics, comorbidities and healthcare utilization throughout the life-course would have provided a more reliable basis for studying the predictors of adverse event mortality. For example, previous evidence has suggested that patients with low socioeconomic status have a higher risk of medical adverse events (Burstin et al., 1992, Stockwell et al., 2019, Shen et al., 2016), but it is also likely that the cause-of-death distribution varies markedly by socioeconomic position, and adverse event deaths may be masked behind higher overall mortality. It would be difficult to reliably distinguish these phenomena from each other using the present dataset.

The strengths of this study include an official data source, nationwide coverage of certified US deaths, and low amount of missing data. The main finding (i.e., the low number of deaths attributed to an adverse event) was clear, and the dataset is publicly accessible online for confirmatory and subsequent analyses. However, the retrospective nature of the dataset, lack of data on the “true” underlying adverse event rate in this population, and lack of follow-up data prevented more sophisticated analysis regarding adverse event mortality.

## 5 Conclusion

This register-based study of 2 846 305 certified deaths attributed 0.16–1.13% of all deaths to an adverse event in the US in 2018. Odds for adverse event death were higher among younger than elderly individuals, among those of black than white ethnicity, and among individuals with higher education level. The present data indirectly support previous evidence that a large number of adverse events remain underrecognized or misclassified. Future analyses are needed to idenfity the root causes behind underreporting and also address the aspect of whether it occurs at random or in a systematic way. A prospective cohort design with detailed data on sociodemographics, comorbidities and healthcare utilization would provide a basis for studying the predictors of adverse event mortality.

## CRediT authorship contribution statement

**Petteri Oura:** Conceptualization, Methodology, Formal analysis, Data curation, Writing – original draft, Writing – review & editing.

## Declaration of Competing Interest

The author declares that he has no known competing financial interests or personal relationships that could have appeared to influence the work reported in this paper.
