# Supplementary material for: Medical adverse events in the US 2018 mortality data
Source: Prev Med Rep. 2021 Oct 2;24:101574. doi: 10.1016/j.pmedr.2021.101574 (PMC8683850; doi:10.1016/j.pmedr.2021.101574)
Supplement: Supplementary data 1 [file mmc1.docx]

**Supplementary Table 1**. Sociodemographic characteristics of adverse event death subtypes with comparisons between subtypes according to underlying-cause data. Values are precentages with frequencies unless otherwise indicated.

| Characteristic | Adverse event subtypes | | | |  | Comparison between subtypes | | | | |  |
| --- | --- | --- | --- | --- | --- | --- | --- | --- | --- | --- | --- |
|  | Complication of medical or surgical procedure without misadventure (n = 4030) | Medication-related adverse event (n = 503) | Medical or surgical misadventure (n = 87) | Medical device-related adverse event (n = 0) |  | OR^1^ (95% CI), P value | | OR^2^ (95% CI), P value | | OR^3^ (95% CI), P value | |
| Sex |  |  |  |  |  |  |  |  |  |  |  |
| Female | 46.1 (1857) | 48.7 (245) | 42.5 (37) | - |  | 1 (Reference) |  | 1 (Reference) |  | 1 (Reference) |  |
| Male | 53.9 (2173) | 51.3 (258) | 57.5 (50) | - |  | 0.90 (0.75—1.09) | 0.284 | **0.64 (0.41—0.98)** | **0.040** | 0.73 (0.45—1.19) | 0.112 |
| Age (years) |  |  |  |  |  |  |  |  |  |  |  |
| < 20 | 2.1 (85) | 0.8 (4) | 4.6 (4) | - |  | 0.59 (0.21—1.69) | 0.329 | 2.10 (0.65—6.75) | 0.212 | 4.20 (0.88—20.1) | 0.073 |
| 20—39 | 3.6 (147) | 6.4 (32) | 4.6 (4) | **-** |  | **2.02 (1.31—3.01)** | **0.002** | 1.73 (0.57—5.26) | 0.332 | 0.89 (0.28—2.89) | 0.849 |
| 40—59 | 16.3 (658) | 22.5 (113) | 13.8 (12) | **-** |  | **1.68 (1.27—2.23)** | **< 0.001** | 1.13 (0.53—2.40) | 0.752 | 0.69 (0.31—1.56) | 0.372 |
| 60—79 | 50.5 (2035) | 47.3 (238) | 57.5 (50) | - |  | 1.12 (0.88—1.41) | 0.366 | 1.58 (0.90—2.77) | 0.109 | 1.38 (0.75—2.54) | 0.302 |
| ≥ 80 | 27.4 (1105) | 23.1 (116) | 19.5 (17) | - |  | 1 (Reference) |  | 1 (Reference) |  | 1 (Reference) |  |
| Unknown | 0 (0) | 0 (0) | 0 (0) | - |  |  |  |  |  |  |  |
| Ethnicity |  |  |  |  |  |  |  |  |  |  |  |
| White | 15.0 (603) | 12.3 (62) | 21.8 (19) | - |  | 1 (Reference) |  | 1 (Reference) |  | 1 (Reference) |  |
| Black | 81.7 (3293) | 84.5 (425) | 70.1 (61) | - |  | 0.77 (0.58—1.02) | 0.072 | 1.58 (0.93—2.68) | 0.091 | **1.90 (1.03—3.51)** | **0.040** |
| Other | 3.3 (134) | 3.2 (16) | 8.0 (7) | - |  | 0.92 (0.54—1.57) | 0.759 | **2.76 (1.23—6.19)** | **0.014** | **2.64 (1.00—6.95)** | **0.049** |
| Education level |  |  |  |  |  |  |  |  |  |  |  |
| High | 19.6 (788) | 19.1 (96) | 18.4 (16) | - |  | 1 (Reference) |  | 1 (Reference) |  | 1 (Reference) |  |
| Medium | 61.5 (2478) | 66.6 (335) | 54.0 (47) | - |  | 1.07 (0.84—1.37) | 0.577 | 0.89 (0.50—1.59) | 0.704 | 0.85 (0.46—1.60) | 0.620 |
| Low | 17.2 (695) | 13.1 (66) | 27.6 (24) | - |  | 0.82 (0.58—1.14) | 0.234 | 1.57 (0.80—3.06) | 0.187 | 1.87 (0.89—3.93) | 0.099 |
| Unknown | 1.7 (69) | 1.2 (6) | 0 (0) | - |  |  |  |  |  |  |  |

CI = Confidence interval, OR = Odds ratio ^1^OR for medication death relative to complication death.
^2^OR for misadventure death relative to complication death.
^3^OR for misadventure death relative to medication death.
